# A Study Assessing the Role of Renal Grayscale Ultrasonography and Flowmetry in Correlation With Renal Function Tests Across Various Renal Diseases

**DOI:** 10.7759/cureus.56681

**Published:** 2024-03-22

**Authors:** Yashaswinii Polaka, Evangeline P Christina, Karthik Krishna Ramakrishnan, Arunkumar Mohanakrishnan, Paarthipan Natarajan

**Affiliations:** 1 Department of Radio-Diagnosis, Saveetha Medical College and Hospital, Saveetha Institute of Medical and Technical Sciences, Saveetha University, Chennai, IND

**Keywords:** acute kidney injury, edv, psv, ultrasound imaging, renal artery doppler

## Abstract

Background

Renal insufficiency, a critical concern in native and transplant kidneys, necessitates effective screening modalities for evaluation and management. Grayscale sonography has been a cornerstone in renal diagnostics, providing basic anatomical insights such as renal length, cortical thickness, and collecting system dilatation. Despite technological advancements, its impact on the differential diagnosis or management of renal disease remains limited, often showing normal findings in the presence of severe renal dysfunction. On the other hand, Doppler sonography, particularly the Doppler resistive index (RI), has shown potential in enhancing the assessment of renal dysfunction by quantifying alterations in renal blood flow and correlating with various renal pathologies and prognoses. Thus, this study aims to assess and compare the sensitivity of transabdominal and Doppler sonography as a diagnostic modality to evaluate medical renal diseases with altered renal function tests (RFTs).

Methodology

Participants included patients visiting the ultrasonography (USG) room at our hospital for USG of the kidneys, ureters, and bladder (USG KUB) and USG of the whole abdomen (USG W/A) with altered RFTs. Each underwent renal grayscale USG and RI measurement, alongside standard RFTs, aiming to investigate the relationship between USG and RI findings and RFT outcomes to assess their predictive accuracy for renal function. Renal grayscale USG assessed parameters including renal dimensions, echogenicity, corticomedullary differentiation, and RI. Data management and charting were conducted with Microsoft Excel 2021 and Microsoft Word 2021. IBM SPSS Statistics for Windows, Version 24 (IBM Corp., Armonk, NY) was utilized for data analysis. The analysis of variance (ANOVA) test examined relationships between renal parameters and RI across diagnostic groups. Furthermore, the chi-square test evaluated associations of renal ultrasound findings with RFTs and their significance.

Results

The study analyzed 93 patients with altered RFTs. Chronic kidney disease (CKD) affected 68 patients, primarily males in their fifth and sixth decades, showing reduced renal dimensions, increased cortical echogenicity, and elevated Doppler RI mean (RIm) with 83% sensitivity. Acute kidney injury (AKI) was found in 12 patients, mainly in their fourth decade, displaying increased renal parameters and elevated RIm with 75% sensitivity. Glomerular diseases, including nephrotic syndrome (NS) and nephritic syndrome (NeS), occurred in 9 patients, predominantly males in their fourth decade, with heightened renal cortical echogenicity and elevated RIm with 55.5% sensitivity. Lupus nephritis (LN) was detected in 4 female patients, despite normal renal parameters, showing elevated serum creatinine levels.

Conclusions

Doppler assessment of renal vascular waveforms effectively identifies chronic renal changes, aiding in the diagnosis of altered RFTs and guiding prognosis. While it detects typical changes like decreased size and parenchymal atrophy, it may not be as indicative of diabetic nephropathy. Key ultrasound indicators such as changes in echotexture and size, along with associated findings like ascites and effusions, help recognize altered renal function and minimize unnecessary interventions.

## Introduction

Grayscale sonography is commonly used as the initial screening method for assessing renal insufficiency in both native and transplant kidneys. However, its diagnostic and management impact on renal disease is often constrained. Despite being introduced in the 1970s, grayscale renal sonography has seen minimal advancements, primarily offering basic anatomical details such as renal length, cortical thickness, and collecting system dilatation [1]. These findings may not accurately reflect significant renal dysfunction, as normal appearances can coexist with substantial renal impairment. For instance, small kidneys may indicate advanced-stage chronic kidney disease (CKD). Common B-mode findings of long-standing kidney disease include decreased renal dimensions, increased cortical echogenicity, reduced visibility of renal pyramids and sinus, marginal irregularities, papillary calcifications, and cysts [1]. Polycystic kidney disease, though rare, can also lead to renal insufficiency with bilaterally enlarged kidneys containing multiple cysts [1,2]

Recent studies suggest that Doppler sonography might enhance the assessment of renal dysfunction. Changes in intrarenal arterial waveforms have been associated with urinary obstruction, various intrinsic renal disorders, and renal vascular disease [1,3]. The Doppler resistive index (RI) has emerged as a valuable parameter for quantifying alterations in renal blood flow linked with renal disease. Elevated RI levels are observed in various kidney diseases and have been correlated with renal function and patient prognosis [1,4]. For example, RI elevation is closely associated with renal arteriolosclerosis and allows for early identification of chronic tubulointerstitial nephritis patients [1,5]. Tubulointerstitial lesions, indicating interstitial fibrosis and loss of tubules and capillaries, are significant histological markers of renal function and long-term prognosis.

Ultrasonography (USG) and color Doppler are widely employed in the initial assessment of renal diseases due to their availability, ease of use, affordability, and lack of adverse side effects. While the USG often aids in diagnosing tubulointerstitial, vascular, and urological pathologies, its effectiveness in medical renal diseases remains somewhat limited [6]. Hence, this study aims to assess and compare the sensitivity of transabdominal and Doppler sonography as a diagnostic tool for evaluating medical renal diseases with altered renal function tests (RFTs).

## Materials and methods

This study, conducted in the Department of Radio-Diagnosis, focused on patients referred for USG examinations, including whole abdomen (W/A), kidneys, ureters, and bladder (KUB), along with renal Doppler studies, all presenting with altered renal function tests (RFTs). Approval for this study was obtained from the Institutional Ethics Committee for Human Research (MC/KOL/IEC/NON-SPON/231/12-2015).

The inclusion criteria comprised patients undergoing USG KUB and W/A with a history of altered RFTs, while the exclusion criteria encompassed individuals with transplanted kidneys, undergoing renal replacement therapy, or presenting with surgical renal diseases, vascular injuries to the kidney, polycystic kidney diseases, or other inherited tubular disorders, as well as those under 20 years of age.

The study utilized transabdominal sonography using the ultrasound machine (Phillips iU22, Philips Healthcare, Amsterdam, The Netherlands) with a C5-2 MHz curvilinear probe, supplemented by a 10 MHz linear transducer when necessary. Patients received bowel preparation for optimal scanning and were positioned supine with arms extended above the head. Longitudinal and transverse scans were conducted over both flanks, with oblique projections facilitating longitudinal renal axis visualization. Renal Doppler utilized continuous wave, color-coded, and pulsed Doppler modes with the C5-2 MHz curvilinear transducer. This involved visualizing the kidney longitudinally, optimizing grayscale and color Doppler parameters, identifying renal artery origins, and obtaining spectral tracings at various kidney segments for velocity measurements.

Biochemical parameters, including proteinuria assessed with urinary dipsticks and RFTs with blood urea and serum creatinine levels, were measured using the Beckman Coulter AU400 analyzer via the Modified Berthelot and Modified Jaffe's methods, respectively, complemented the imaging techniques for comprehensive evaluation.

Values were obtained using renal grayscale USG, and parameters, including renal dimensions, echogenicity, and RI, were evaluated. All the data sheet maintenance and charting were done using Microsoft Excel 2021 and Microsoft Word 2021. Data analysis was executed using IBM SPSS Statistics for Windows, version 24 (IBM Corp., Armonk, NY). The analysis of variance (ANOVA) test was used to discern the relationship between renal parameters (such as dimensions, and echogenicity) and RI across different diagnostic groups. Additionally, the chi-square test was applied to evaluate the association of renal ultrasound findings with RFTs and their significance of the association.

A significance level of *P *< 0.05 was considered statistically significant. This study's design and methodology provide a comprehensive framework for analyzing different renal pathologies. By employing rigorous imaging protocols and statistical analyses, the study aims to contribute valuable insights into the understanding of the RI and its correlation with RFTs.

## Results

The study included 93 patients exhibiting altered renal function tests, primarily characterized by elevated serum urea and creatinine levels. Relevant clinical and laboratory information, along with ultrasound characteristics such as Doppler waveforms and RI were evaluated and recorded. The study population was divided into four subgroups for analysis: CKD, acute kidney injury (AKI), glomerular diseases (nephritic syndrome [NeS] and nephrotic syndrome [NS]), and lupus nephritis (LN). Results were organized and presented in tables and charts categorized under different headings, including demographic profiles, ultrasound features, and clinical features.

Among the 93 patients, 60 (62.37%) were male and 33 (37.63%) were female, resulting in a male-to-female ratio of 1.8:1. The mean ages at presentation for males and females were 54.71 and 47.03 years, respectively. The majority of cases were observed in individuals aged between the fourth and sixth decades of life (Table 1).

**Table 1:** Age and sex distribution of the study population.

Age group (in years)	Male sex, *n* (%)	Female sex, *n *(%)	Total, *n* (%)
20-29	2 (2.15%)	5 (5.38%)	7 (7.53%)
30-39	10 (10.75%)	11 (11.83%)	21 (22.58%)
40-49	9 (9.68%)	3 (3.23%)	12 (12.90%)
50-59	13 (13.98%)	4 (4.30%)	17 (18.28%)
60-69	21 (22.58%)	6(6.45%)	27 (29.03%)
>70	5 (5.38%)	4(4.30%)	9 (9.68%)
Total	60 (64.52%)	33(35.48%)	93 (100%)

For this study, CKD was defined according to the Kidney Disease Outcomes Quality Initiative (KDOQI) criteria, which categorize CKD into five stages based on glomerular filtration rate (GFR) and/or markers of kidney damage. These stages range from Stage 1, characterized by kidney damage with normal or increased GFR (>90 mL/min/1.73 m²), to Stage 5, representing kidney failure (GFR < 15 mL/min/1.73 m²) or end-stage renal disease (ESRD) requiring renal replacement therapy (dialysis or transplantation).

There were 68 (73.12%) patients with CKD, 13 (12.90%) patients with AKI, 3 (3.23%) patients with glomerular diseases (NeS), 6 (6.45%) patients with glomerular diseases (NS), and 4 (4.3%) patients with LN. The majority of patients belonged to the CKD group, followed by the AKI group. The distribution of diseases is detailed in Table 2.

**Table 2:** Disease-wise distribution of the study population. CKD, chronic kidney disease; AKI, acute kidney injury; NeS, nephritic syndrome; NS, nephrotic syndrome; LN, lupus nephritis

Disease	Number, *n* (%)
CKD	68 (73.12%)
AKI	12 (12.90%)
NeS	3 (3.23%)
NS	6 (6.45%)
LN	4 (4.03%)
Total	93 (100%)

Among the 68 patients with CKD, 48 (70.58%) were males and 20 (29.42%) were females, indicating a clear male predominance with a male-to-female ratio of 2.4:1. Patients under 20 years of age were excluded from the study. The overall mean age at presentation for CKD was 52.28 (mean ± SD = 52.28 ± 15.78) years. The mean age for males was 55.48 years, while for females, it was 46.97 years (Table 3).

**Table 3:** Age and sex distribution of CKD. CKD, chronic kidney disease

Age group (in years)	Male sex, *n* (%)	Female sex, *n *(%)	Total, *n* (%)
20-29	0	1 (1.08%)	1 (1.47%)
30-39	4 (4.3%)	5 (5.38%)	9 (13.24%)
40-49	7 (7.53%)	2 (2.15%)	9 (13.24%)
50-59	11 (11.83%)	4 (4.30%)	15 (22.06%)
60-69	21 (22.58%)	5 (5.38%)	26 (38.24%)
>70	5 (5.38%)	3 (3.23%)	8 (11.76%)
Total	48 (51.61%)	20 (21.51%)	68 (100%)

For this study, AKI will be operationally defined as a sudden impairment of kidney function characterized by an increase in blood urea nitrogen (BUN) and/or plasma or serum creatinine concentration, often accompanied by a reduction in urine volume. AKI encompasses a heterogeneous group of conditions that can range in severity from asymptomatic and transient changes in laboratory parameters and GFR to overwhelming and rapidly fatal derangements in circulatory volume regulation, electrolyte concentrations, and acid-base composition of plasma. 

Among the 12 patients with AKI, 7 (58.33%) were females and 5 (41.66%) were males, indicating a clear female predominance, with a female-to-male ratio of 1.4:1. Patients under 20 years of age were excluded from the study. The overall mean age at presentation for AKI was 42 (mean ± SD = 42 ± 14.50) years. The mean age for males was 38.8 years, while for females, it was 44.29 years (Table 4).

**Table 4:** Age and sex distribution of AKI. AKI, acute kidney injury

Age group (in years)	Male sex, *n* (%)	Female sex, *n* (%)	Total, *n* (%)
20-29	1 (1.08%)	1 (1.08%)	2 (16.67%)
30-39	2 (2.15%)	3 (3.23%)	5 (41.64%)
40-49	1 (1.08%)	1 (1.08%)	2 (16.67%)
50-59	1 (1.08%)	0	1 (8.33%)
60-69	0	1 (1.08%)	1 (8.33%)
>70	0	1 (1.08%)	1 (8.33%)
Total	5 (5.38%)	7 (7.53%)	12 (100%)

Eight patients had glomerular disease, with 6 diagnosed with acute NeS and 2 with NS, showcasing a clear male predominance with a male-to-female ratio of 2:1. Patients aged 20 years and older were included in the study. The overall mean age at presentation for glomerular disease was 42.23 (mean ± SD = 42.23 ± 14.15) years. The mean age for NeS was 43 years, while for NS, it was 34.5 years. The majority of cases occurred in the fourth decade of life, as depicted in Table 5.

**Table 5:** Age and type distribution of glomerular diseases. NeS, nephritic syndrome; NS, nephrotic syndrome

Age group (in years)	NeS type, *n* (%)	NS type, *n* (%)	Total, *n* (%)
20-29	1 (1.08%)	0	1 (11.11%)
30-39	3 (3.23%)	3 (3.23%)	6 (66.67%)
40-49	1 (1.08%)	0	1 (11.11%)
50-59	1 (1.08%)	0	1 (11.11%)
60-69	0	0	0
>70	0	0	0
Total	5 (5.38%)	3 (3.23%)	12 (100%)

Among the 93 patients, renal dimensions and cortical thickness were evaluated. For the left kidney, 18 (19.35%) patients exhibited reduced renal length, 60 (64.52%) showed normal length, and 15 (16.13%) displayed increased length. Similarly, for the right kidney, 17 (18.28%) patients had reduced renal length, 61 (65.59%) had normal length, and 15 (16.13%) had increased length, as indicated in Table 6. Concerning cortical thickness, 18 (19.35%) patients had reduced thickness, 60 (64.52%) had normal thickness, and 15 (16.13%) had increased thickness for the left kidney. Likewise, for the right kidney, 17 (18.28%) patients had reduced cortical thickness, 61 (65.59%) had normal thickness, and 15 (16.13%) had increased cortical thickness, as depicted in Table 6. The normal ranges considered were 8-12 cm for renal length and 1.5-3 cm for renal cortical thickness, as outlined in Table 6.

**Table 6:** Disease-wise distribution of the renal length and cortical thickness. CKD, chronic kidney disease; AKI, acute kidney injury; NeS, nephritic syndrome; NS, nephrotic syndrome; LN, lupus nephritis

Disease	Left renal length				Right renal length				Left cortical thickness				Right cortical thickness			
	Decreased (*n*, %)	Normal (*n*, %)	Increased (*n*, %)	Mean length (cm)	Decreased (*n*, %)	Normal (*n*, %)	Increased (*n*, %)	Mean length (cm)	Decreased (*n*, %)	Normal (*n*, %)	Increased (*n*, %)	Mean length (cm)	Decreased (*n*, %)	Normal (*n*, %)	Increased (*n*, %)	Mean length (cm)
CKD	18 (19.35%)	43 (46.24%)	7 (7.53%)	9.34	17 (18.28%)	44 (47.31%)	7 (7.53%)	9.41	18 (19.35%)	43 (46.24%)	7 (7.53%)	2.13	17 (18.28%)	44 (47.31%)	7 (7.53%)	2.28
AKI	0	6 (6.45%)	6 (6.45%)	12.41	0	6 (6.45%)	6	12.11	0	6 (6.45%)	6 (6.45%)	3.11	0	6 (6.45%)	6 (6.45%)	3.95
NeS	0	3 (3.23%)	0	10.25	0	3 (3.23%)	0	9.73	0	3 (3.23%)	0	2.36	0	3 (3.23%)	0	2.33
NS	0	4 (4.30%)	2 (2.15%)	10.73	0	4 (4.30%)	2 (2.15%)	10.36	0	4 (4.30%)	2 (2.15%)	2.66	0	4 (4.30%)	2 (2.15%)	2.03
LN	0	4 (4.30%)	0	11.11	0	4 (4.30%)	0	11.11	0	4 (4.30%)	0	2.93	0	4 (4.30%)	0	2.70
Total	18 (19.35%)	60 (64.52%)	15 (16.13%)	9.79	17 (18.28%)	61 (65.59%)	15 (16.13%)	9.81	18 (19.35%)	60 (64.52%)	15 (16.13%)	2.10	17 (18.28%)	61 (65.59%)	15 (16.13%)	2.24

In the study, renal cortical echogenicity was assessed using a grading system based on comparison with the echogenicity of the liver and renal sinus. This grading system consists of four groups: Grade 0 represents a normal condition where the echogenicity of the right renal cortex is observed to be less than that of the liver. In Grade I, the echogenicity of the right renal cortex equals that of the liver. Grade II indicates a higher echogenicity of the right renal cortex compared to the liver but remains less than that of the renal sinus. Finally, Grade III signifies that the echogenicity of the right renal cortex is equivalent to that of the renal sinus.

**Figure 1:**
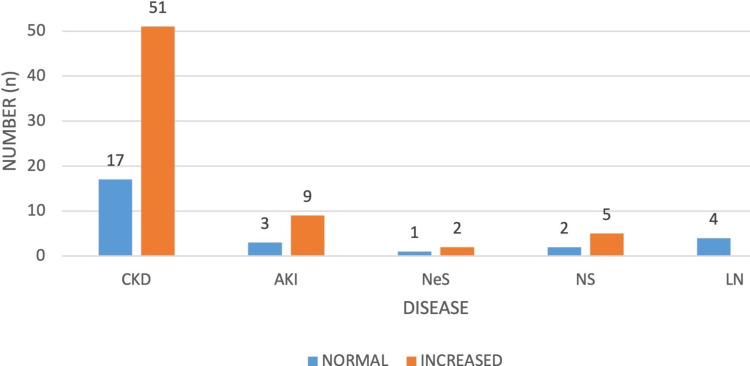
Disease-wise distribution of renal echotexture in relation to serum creatinine levels. CKD, chronic kidney disease; AKI, acute kidney injury; NeS, nephritic syndrome; NS, nephrotic syndrome; LN, lupus nephritis

It revealed 29 (31.18%) patients with normal echotexture and 64 (68.82%) patients with increased echotexture bilaterally, predominantly observed in cases of CKD. Specifically, among the 68 (73.12%) CKD patients, 17 (18.28%) had normal echotexture, while 51 (54.84%) exhibited increased echotexture, with varying grades noted. In AKI, 9 out of 12 patients had increased echotexture, distributed as 2 (2.15%) in Grade I and 7 (7.53%) in Grade II. Notably, all LN patients displayed normal echotexture. Among the 9 (9.68%) patients with glomerular diseases, 6 (6.45%) had increased echotexture, with 4 (4.30%) in Grade II and 2 (2.15%) in Grade III (Figure 1).

**Figure 2:**
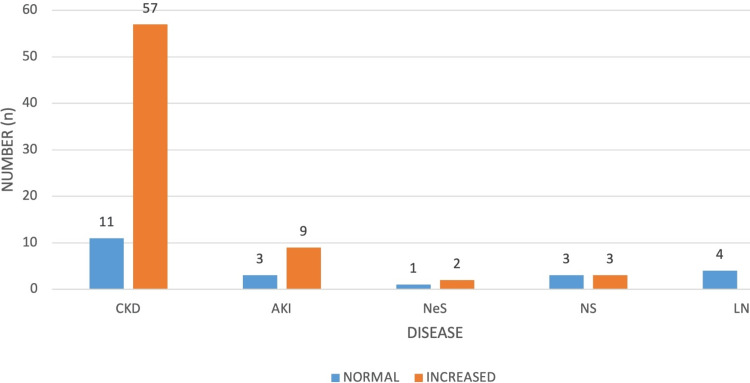
Disease-wise distribution of RI in both kidneys. RI, resistivity index; CKD, chronic kidney disease; AKI, acute kidney injury; NeS, nephritic syndrome; NS, nephrotic syndrome; LN, lupus nephritis

Among 93 patients, 22 (23.66%) showed a normal RI (<0.7), while 71 (76.34%) patients exhibited increased RI in both kidneys. The majority of cases were observed in CKD. The disease-wise distribution of the renal interlobar artery RI is depicted in Figure 2.

**Figure 3:**
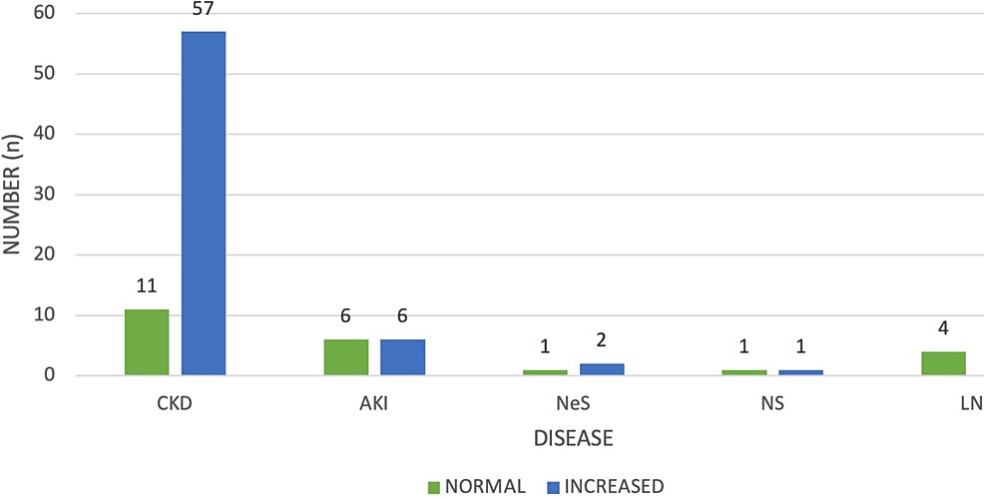
Disease-wise distribution of PI in both kidneys. PI, pulsatility index; CKD, chronic kidney disease; AKI, acute kidney injury; NeS, nephritic syndrome; NS, nephrotic syndrome; LN, lupus nephritis

Among 93 patients, 21 (22.58%) showed a normal pulsatility index (PI), while 72 (76.34%) patients exhibited an increased PI in both kidneys. The majority of cases were observed in CKD. The disease-wise distribution of the PI is shown in Figure 3. Normal PI is defined as <1.2.

**Figure 4:**
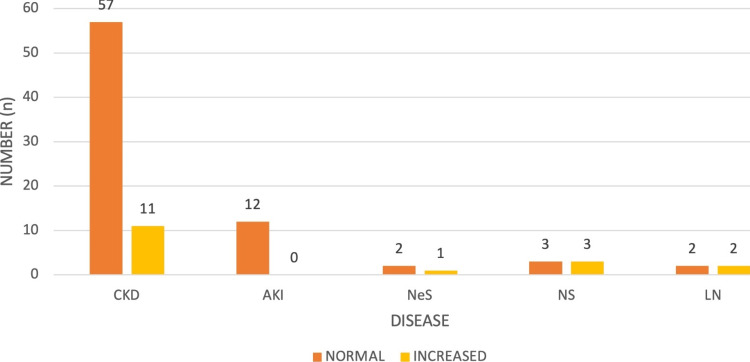
Disease-wise distribution of PSV in both kidneys. PSV, peak systolic velocity; CKD, chronic kidney disease; AKI, acute kidney injury; NeS, nephritic syndrome; NS, nephrotic syndrome; LN, lupus nephritis

Among 93 patients, 72 exhibited a normal peak systolic velocity in the aorta (PSVa), while 21 patients showed increased PSVa. The majority of cases were observed in CKD. The disease-wise distribution of PSVa is depicted in Figure 4. Normal PSVa falls within the range of 60-100 cm/s.

**Figure 5:**
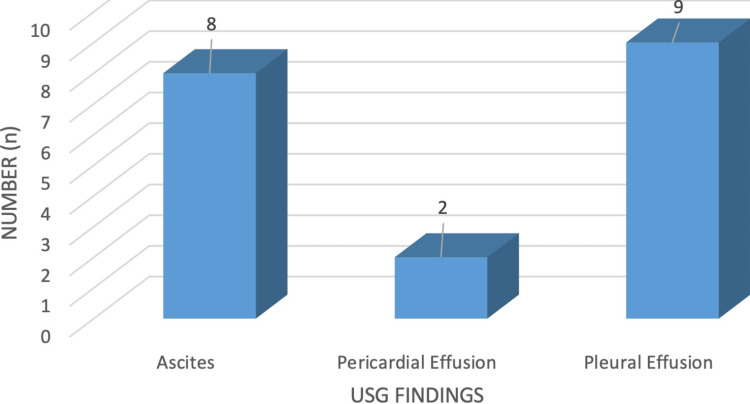
Miscellaneous: other USG findings. USG, ultrasonography

Among 93 patients, 8 (8.60%) patients had ascites (7 with CKD and 1 with NS), 2 (2.15%) had pericardial effusions (CKD), and 9 (9.68%) had pleural effusions (CKD), as shown in Figure 5.

**Figure 6:**
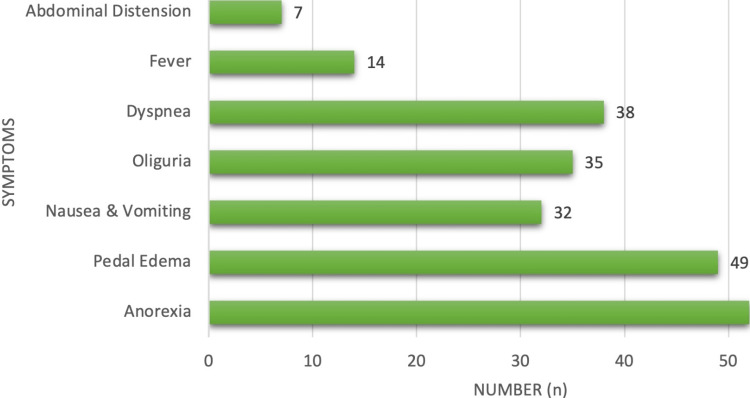
Clinical features in chronic kidney diseases.

The most common presenting features in CKD were anorexia in 52 patients (76.47%) and pedal edema in 49 patients (72.06%), followed by dyspnea in 38 cases (55.88%), oliguria in 35 (51.47%), nausea/vomiting in 32 cases (47.06%), and abdominal distension in 7 cases (10.29%) (Figure 6).

**Figure 7:**
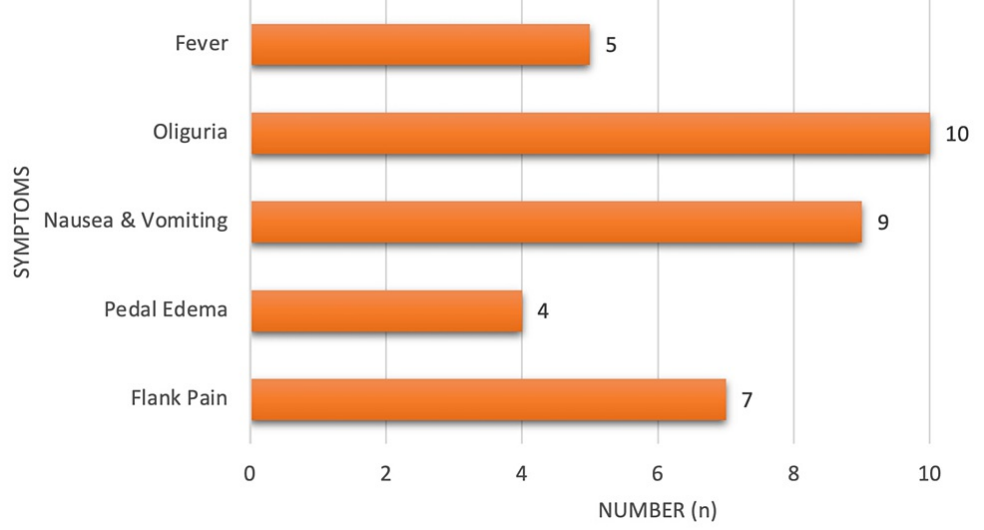
Clinical features in acute kidney injury.

The most common presenting features in AKI were oliguria in 10 cases (88.33%) and nausea/vomiting in 9 patients (75%), followed by fever in 5 patients (41.67%), flank pain in 7 cases (58.33%), and pedal edema in 4 cases (33.33%) (Figure 7).

**Figure 8:**
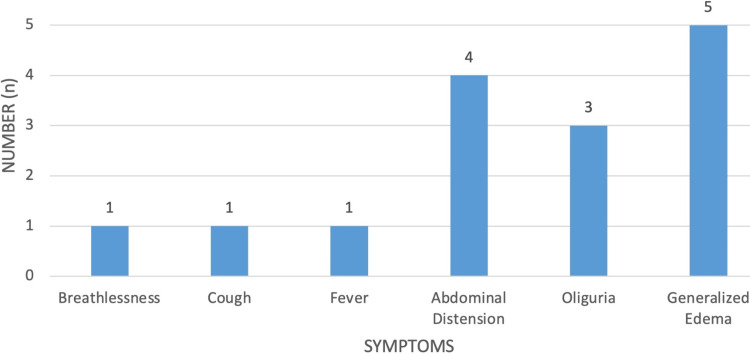
Clinical features in nephrotic syndrome.

The most common presenting features in NS were pedal edema in 5 patients (83.33%) and abdominal distension in 4 patients (66.67%), followed by oliguria in 3 cases (50%), breathlessness in 1 case (16.67%) and cough in 1 case (16.67%) (Figure 8).

**Figure 9:**
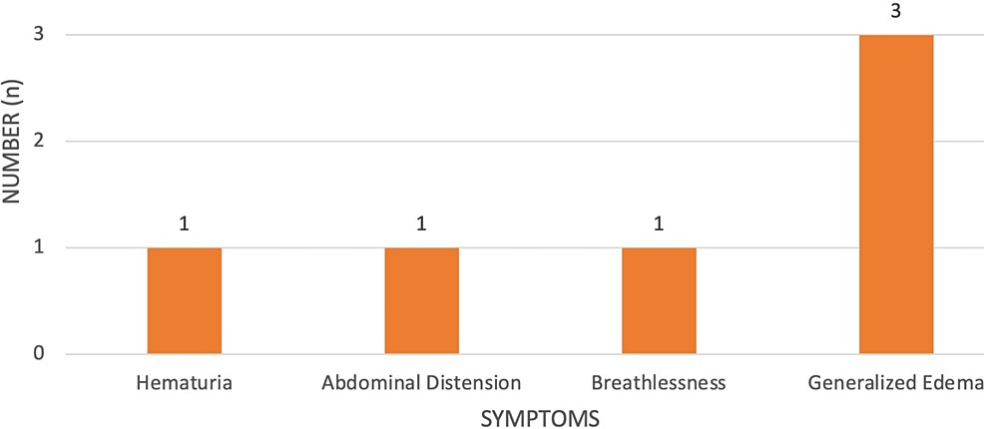
Clinical features in nephritic syndrome.

The most common presenting features in NeS were pedal edema in 3 patients (100%), followed by hematuria (1, 33.3%), breathlessness (1, 33.3%), and abdominal distension (1, 33.3%), as shown in Figure 9.

**Figure 10:**
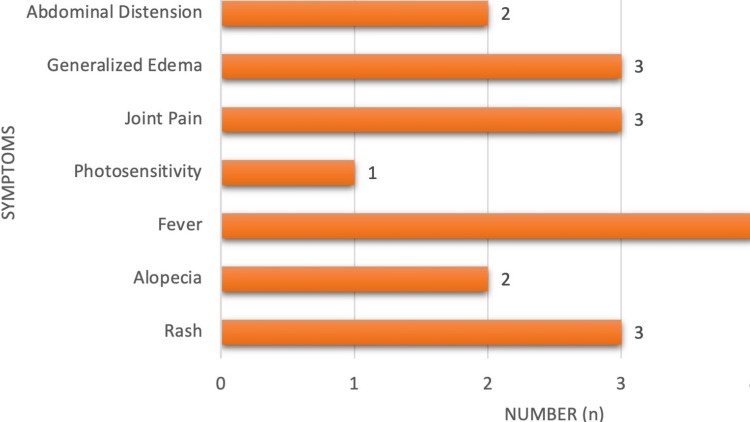
Clinical features in lupus nephritis.

The most common presenting features in LN were fever in all patients (100%) followed by joint pain (3, 75%), generalized edema (3, 75%), rash (3, 75%), abdominal distension (2, 50%), alopecia (2, 50%), and photosensitivity (1, 25%), as shown in Figure 10.

## Discussion

The study, conducted in the Department of Radio-Diagnosis, involved 93 patients exhibiting altered RFTs, primarily elevated serum urea and creatinine levels. Doppler sonography was employed to assess these cases, focusing on USG characteristics and Doppler waveforms. The mean age of presentation was 51.98 years, with a notable male predominance in CKD cases. These findings align closely with existing literature, with studies by Agarwal and Light and Viswanathan et al. showing similar age distributions for CKD, albeit with minor regional variations [7,8]. 

In our study, the male-to-female ratio in CKD was 2.4:1, consistent with findings by Moranne et al., where it was reported as 2.22:1 [9]. Regarding LN, the mean age of presentation was 28.25 years in our study, aligning with Dhir et al.'s findings of 23.6 years for CKD presentation, albeit with a male-to-female ratio of 11:1, attributed to our study's inclusion of only female patients, possibly due to small sample size [10]. Additionally, the mean age of presentation for AKI in our study was 41.23 years, in contrast to Liaño and Pascual's study where it was 63 years, suggesting potential regional variations in age incidence [11].

Renal size and cortical thickness varied, with 18 patients showing reduction, 43 displaying normal values, and 7 presenting increased size on the left side, mirroring similar findings on the right side. Notably, increased kidney size and cortical thickness correlated with certain renal conditions like NS, NeS, and AKI, as described in standard pathology texts. Diabetic nephropathy accounted for 31.2% of CKD cases in India, with asymptomatic cases exhibiting enlarged kidneys, aligning with our observations. Studies by Platt et al. [12] and Hricak et al. [13] highlighted the limitations of using renal echogenicity alone for diagnosis, emphasizing the need for a comprehensive assessment. Among CKD patients, 51 exhibited increased renal cortical echotexture, with grades correlating significantly with serum creatinine levels [12]. Similar correlations were found in AKI and glomerular diseases [12,13]. However, in LN, all patients had normal cortical echotexture, precluding further analysis. These findings underscore the potential of renal cortical echogenicity grading in aiding diagnosis and prognosis across various renal disorders. By using a one-way ANOVA test, the *P*-value was found to be 0.032, which was significant, suggesting that serum creatinine was statistically related to grading based on cortical echogenicity in glomerular diseases, which correlated with the study by Tsau et al. In LN, the ANOVA could not be calculated as all patients had normal (Grade 0) cortical echotexture [14]. Hypertension was prevalent among patients with CKD and glomerular disease, in line with findings from Soni et al., although slight variations in incidence rates were noted, potentially due to regional differences [15].

In our CKD study, RIm was 0.83, contrasting with 0.59 in Izumi et al.'s outpatient-focused research, likely due to different patient populations [16]. Our AKI RIm sensitivity at 0.75 aligned closely with Bossard et al's findings (0.85) [17]. Interestingly, LN patients showed no increase in RIm, consistent with Ozbek et al.'s conclusion on the limited utility of Doppler ultrasound in early LN. For glomerular diseases (NeS and NS), our sensitivity was 55%, slightly lower than Izumi et al.'s [16] and Ozbek et al.'s 61.9% [18].

The common presenting features in our study included anorexia in 71 (76.7%), pedal edema in 67 (72%), dyspnea in 52 (55.88%), oliguria in 48 (51.4%), and nausea/vomiting in 44 (47%). Breathlessness in 51 (54.4%) and generalized edema in 78 (83.3%) were also prevalent. Similarly, fever in 93 (100%), joint pain in 70 (75%), and rashes in 70 (75%) were commonly reported symptoms. Additionally, ascites were observed in 8 (8.60%) patients, pericardial effusion in 2 (2.15%), and pleural effusion in 9 (9.68%), consistent with findings from previous studies by Ackerman [19] in CKD patients.

Overall, the study's findings align well with existing literature, highlighting the utility of Doppler sonography in evaluating renal function and pathology. These observations contribute to the growing body of evidence supporting the use of renal USG as a valuable tool in the diagnosis and management of various renal disorders.

## Conclusions

Doppler assessment of renal vascular waveforms can effectively identify abnormalities in RFTs associated with various medical renal diseases. Many chronic renal conditions ultimately result in reduced renal size, parenchymal atrophy, sclerosis, and fibrosis, as observed through ultrasound imaging showing smaller kidneys, parenchymal thinning, hyperechogenicity indicative of sclerosis and fibrosis, and secondary cystic changes. Notably, diabetic nephropathy, a leading cause of chronic and end-stage renal failure globally and in countries like India, often does not exhibit these typical ultrasound findings. Doppler evaluation of intrarenal vessels offers additional insights into microvascular and parenchymal lesions, aiding in treatment decisions and timely planning for optimal renal replacement therapy. While renal color Doppler ultrasound cannot currently differentiate between different types of medical renal disorders and their etiologies, it serves to identify irreversible diseases, assess prognosis, and prevent unnecessary diagnostic or therapeutic procedures. Specific intrarenal ultrasound features like increased echotexture and irregular renal surface, along with associated extrarenal features such as ascites, pleural effusion, and pericardial effusion, can serve as valuable indicators of altered renal function.
